# Evolution of lattice distortions in 4H-SiC wafers with varying doping

**DOI:** 10.1038/s41598-020-67900-y

**Published:** 2020-07-02

**Authors:** Nadeemullah A. Mahadik, Hrishikesh Das, Stanislav Stoupin, Robert E. Stahlbush, Peter L. Bonanno, Xueping Xu, Varatharajan Rengarajan, Gary E. Ruland

**Affiliations:** 1 US Naval Research Laboratory, Washington, DC USA; 2 On Semiconductor, South Portland, ME USA; 3 Cornell High Energy Synchrotron Source, Ithaca, NY USA; 4 II-VI Advanced Materials, Pine Brook, NJ 07058 USA

**Keywords:** Materials science, Condensed-matter physics, Materials for devices, Applied physics, Characterization and analytical techniques, Design, synthesis and processing, Imaging techniques, Microscopy

## Abstract

Lattice distortions (LD) in 4H-silicon carbide (SiC) wafers were quantified using synchrotron X-ray rocking curve mapping (RCM), and were resolved into their two components of lattice strain (Δd/d) and lattice plane curvature (LPC) for 150 mm diameter wafers. The evolution of these LDs were investigated for three sequential substrates from the same boule, one of which was the substrate reference, and the other two had a 10 µm thick, 1 × 10^17^ and 4 × 10^14^ cm^-3^ n-type doped epitaxial layer. The lattice strain, Δd/d, was highest for the lowest doped wafer due to higher mismatch with the substrate wafer. After epitaxial layer growth, the LPC variation across the wafer increases by a factor of 2, irrespective of doping. The LPC maps indicate presence of a twist in the lattice planes that increases after epitaxial growth. The LPC component has higher influence on wafer shape change, which can reduce device yields. The lattice strain component predominantly affects the glide of basal plane dislocations (BPDs), thereby reducing device reliability. From analysis of peak widths, it was determined that threading dislocations in the top 6 microns of the wafer increase after epitaxial layer growth.

## Introduction

Silicon Carbide (SiC) based power devices are a small but rapidly growing segment of the Si dominated power electronics market^1,2^. Adoption of the SiC devices has been increasing rapidly, but is still limited by cost and less well established reliability^3–5^. The presence and evolution of strain in SiC wafers during epitaxial growth and device fabrication pose both cost and reliability concerns. Following the SiC substrate growth by the non-equilibrium, physical vapor transport technique (PVT), SiC substrates have residual strain and lattice distortions (LD) that varies throughout the boule. During epitaxial layer growth or device fabrication steps, the substrate LD can cause wafer shape changes, and the evolution of these distortions has not yet been quantified. The device yield concern is that the wafer shape changes driven by LD can result in unpredictable wafer shape change. Excessive LDs can cause failure in fabrication steps such as lithography problems from inability to accommodate the wafer curvature in mask steppers, and wafer damage from handling of distorted wafers, which suppresses net device yield.

The strain from LDs is responsible for the stress that causes the basal plane dislocations (BPD) in SiC epitaxial layers to glide and to affect a larger wafer area. For example, a single gliding BPD can create an extended defect such as a half-loop array (HLA) that will affect any device along its path. During device operation, they generate stacking faults that expand and degrade the conductivity of the device drift layer^6–9^. The BPDs have also been shown to generate stacking faults in unipolar devices by stressing the body diode during switching^10,11^. Hence, BPD glide is often detrimental to device reliability and performance.

Lattice distortions have two components namely, lattice strain (Δd/d), and lattice plane curvature (LPC). They arise from different origins in the SiC wafers such as residual strain in the substrates. For both substrates and epitaxial layers LDs can arise from doping variation, thermal stress, and extended defect concentrations. It is very important to quantify them separately in order to evaluate how the different origins influence extended defects and device yield. Additionally, how the two LD components evolve with epitaxial layer growth and doping has not been systematically investigated. This is important to understand which component varies and can lead to adverse effects in device fabrication and reliability. In this work, lattice distortions are measured by using synchrotron X-ray rocking curve mapping (RCM) technique^12^, which measures X-ray Bragg peak variation over the entire SiC wafers. The two LD components are explained below; along with how they both affect the Bragg peak variation, and how they can be separately quantified.

### Two types of lattice distortions

**Figure 1:**
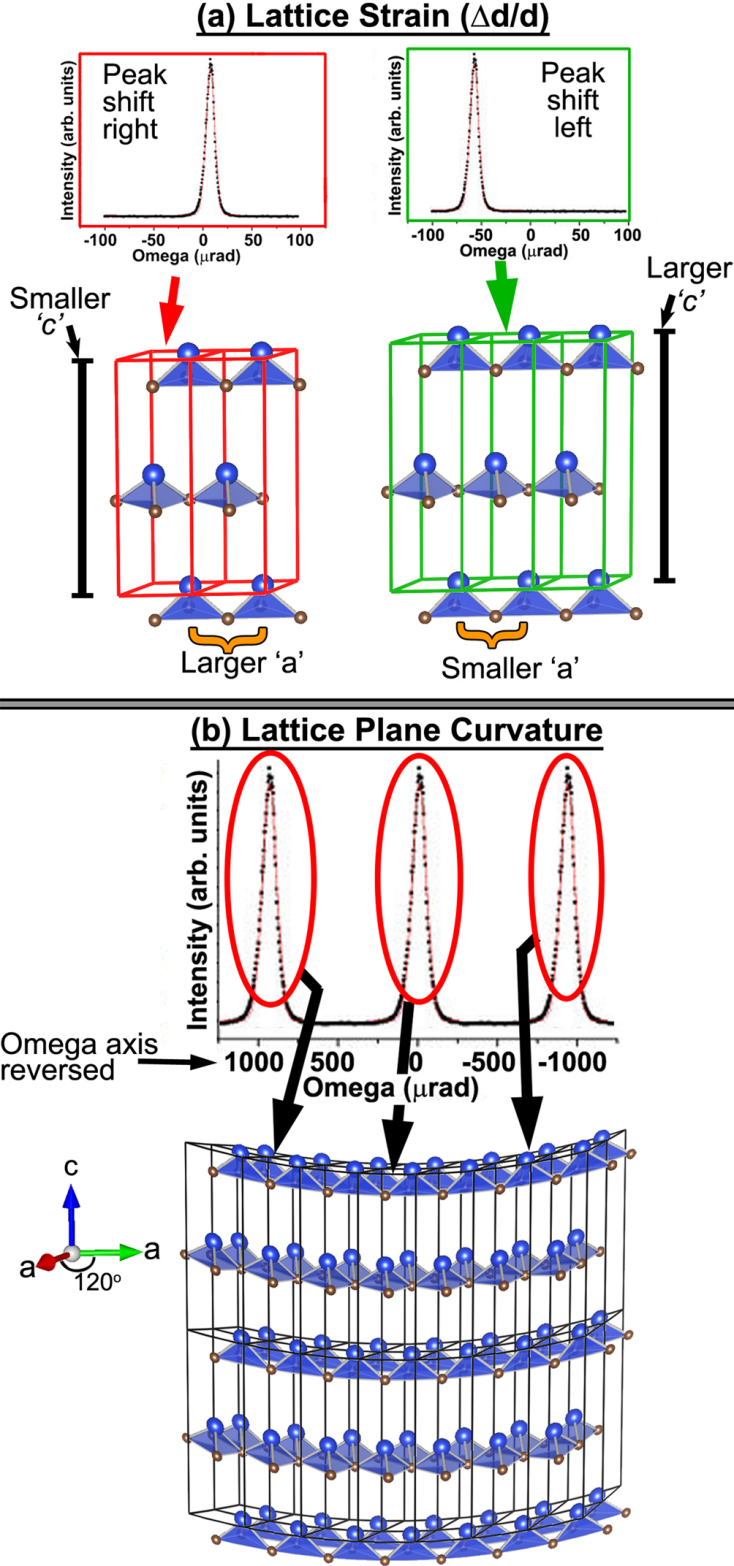
Schematics showing the two types of lattice distortions in the 4H SiC lattice and their effect on the X-ray rocking curve: (**a**) lattice strain, Δd/d, and (**b**) lattice plane curvature (LPC). These figures were generated by the authors using VESTA software ver. 3.4.6. The software is distributed free for scientific use.

Strain in crystalline materials has been measured by using X-ray diffraction measurements for many decades. The crystalline planes diffract the incident X-rays producing peaks at the Bragg scattering angle. The diffracted peaks from different regions in a wafer will shift by the angle *‘*Δ*w*’, and this shift is caused by the lattice parameter variation, also known as, the lattice strain, ‘Δ*d/d*’ and by the lattice plane curvature (LPC) according to the relation^13^,

1 $$\Delta w=\mathrm{tan}{\theta }_{B}\Delta d/d+\left(\widehat{{n}_{r}}\bullet \widehat{{n}_{m}}\right)\Delta \rho$$

where $\theta$_*B*_ is the reference Bragg angle. The reference point on the wafer is taken from the median point of the X-ray peak positions collected. The unit vector, ${\widehat{n}}_{r}$, is the direction of the rocking curve rotation axis, ${\widehat{n}}_{m}$ is the direction of the tilt axis, and Δ*ρ* is the tilt angle. Hence, at the reference point the dot product ${\widehat{n}}_{r}\bullet {\widehat{n}}_{m}$ is 1. The first term of Eq. (1) is the contribution to $\Delta w$ due to the lattice strain and the second term is the contribution due to LPC. The illustration in Fig. 1a show five 4H-SiC unit cells along the *a*-axis. The illustration in Fig. 1b shows two rows of 4H-SiC unit cells stacked along the *c*-axis (2 layers each), and 7 unit cells along the *a*-axis thereby showing a small ensemble of the 4H-SiC structure. These two small ensembles illustrate how the two LD components will cause peak shifts in the X-ray RCM measurements, and how they can be directly measured. In Fig. 1a, the five 4H-SiC unit cells are drawn in two groupings, with artificial space between them for clarity. The *‘a’* and *‘c’* lattice parameters are marked. These two groupings have different *a*, and *c* lattice parameters that can be caused by variation in epitaxial layer doping. The unit cells are scaled in order to maintain the Poisson’s ratio of 0.18 in the 4H-SiC hexagonal structure^14^. This means when the *a-* parameter is enlarged, the *c-* parameter is reduced, and vice versa. When symmetric X-ray Bragg reflections are measured along the *c*-plane, the 2 different spectra shown above the unit cell groupings illustrate how the X-ray peak shifts will occur due to the lattice strain, Δd/d. A smaller *c-*parameter will shift the peak to higher angles, whereas larger *c-*parameter will cause a peak shift to lower angles. This lattice strain can have origins due to doping variations, and thermal stress^15–17^.

In Fig. 1b, a similar unit cell lattice structure of 4H-SiC is depicted with a lattice plane curvature (bow/warp). The LPC originates from residual strain from substrate growth and extended defects, such as dislocations, in both substrate and epitaxial layers. It can be observed from the schematic in Fig. 1b, the curvature at a local region is influenced by forces exerted by adjacent regions. This is different from the Δd/d, which is a dilation/compression from within the lattice. The lattice curvature causes the diffracted X-rays peaks to shift corresponding to the local slope of the curvature. Notice that the range of peak shift (x-axis) for the LPC component is drawn to be ~ 10 × larger than the x-axis range in Fig. 1a. This is expected in typical SiC wafers. In case of Fig. 1b, the x-axis is also plotted from positive to negative for figure clarity. The LPC variation can lead to wafer misalignment during lithography. In order to precisely quantify the components of LD in this work, we have performed a systematic investigation of LD evolution in a series of same boule adjacent wafers with and without epitaxial layers having varying doping. This includes determining the LD contributions from Δd/d and lattice plane curvature.

In order to deconvolve the two LD components, we have used the RCM technique described by Bonse^18^ and Kikuta et al.^19^, where the same Bragg condition peak shifts, Δw, across a region in a wafer are collected at two azimuthal conditions that are mutually 180° apart. In this case, the normal vector in Eq. (1) changes sign and we obtain the two LD components using the relations:

2 $$\Delta {w}_{{0}^{o}}=\mathrm{tan}{\theta }_{B}\Delta d/d+LPC$$

3 $$\Delta {w}_{{180}^{o}}=\mathrm{tan}{\theta }_{B}\Delta d/d-LPC$$

Taking the sum and difference of eqns. (2) and (3), the two components of strains can be independently computed. This technique has been used to precisely measure the origin of strains in a variety of substrates and epitaxial layers^20–24^.

## Results and discussion

**Figure 2:**
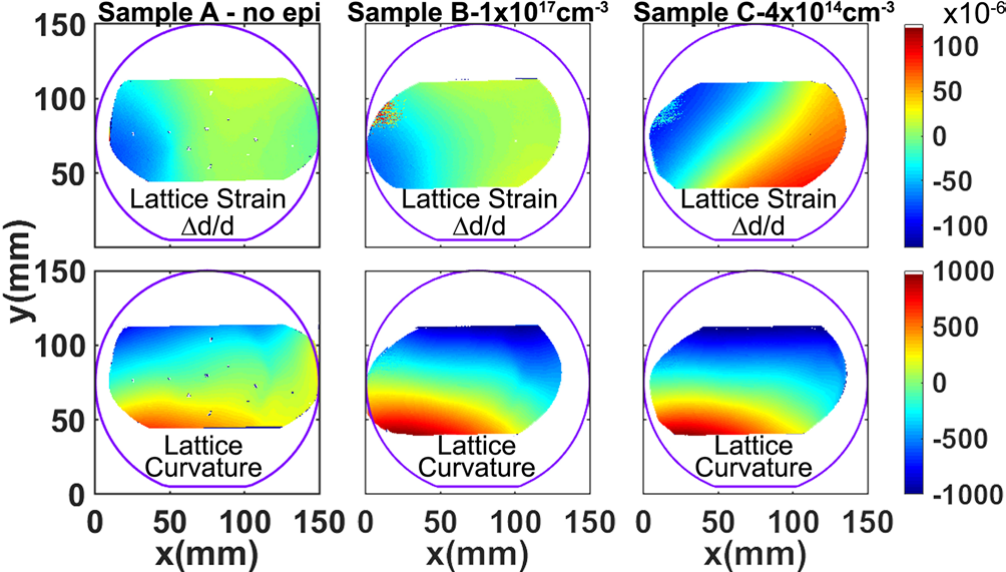
Wafer maps of the two components of lattice distortions, (top row) lattice strain, Δd/d, and (bottom row) lattice plane curvature, LPC, deconvolved from X-ray data collected at two azimuthal positions 180° apart for samples **A**, **B**, and **C**. This figure was generated using 2018 Matlab software with current license purchased by the authors.

**Figure 3:**
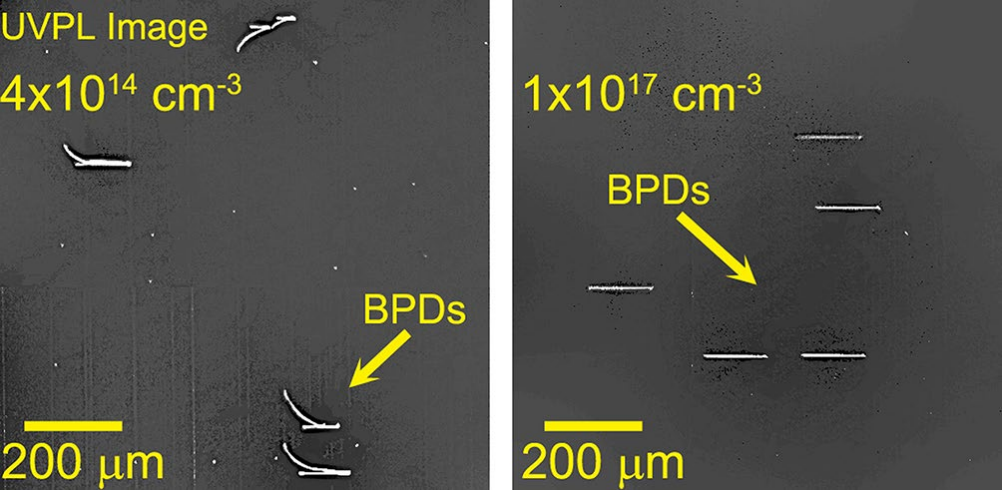
UVPL images of regions from low-doped, sample **C** (left) showing curved BPDs and high doped, sample **B** (right) epitaxial layers showing straight BPDs.

**Figure 4:**
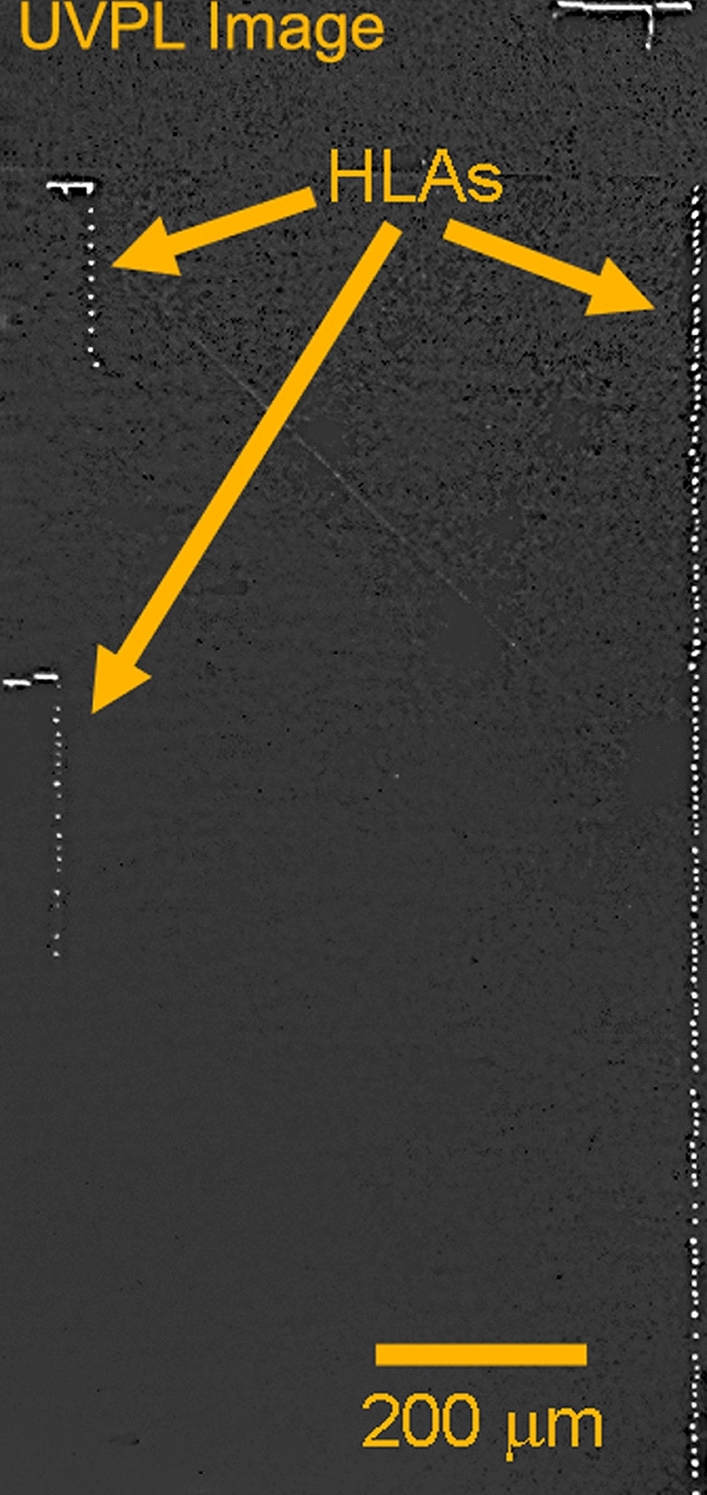
UVPL image of an epitaxial layer having HLAs showing the BPD glide occurring over a larger region of the sample.

Lattice strain (Δd/d) maps and LPC maps were generated for the three SiC sequential wafers labeled sample A: substrate only, sample B: epitaxial layer with 1 × 10^17^ cm^-3^ n-type doping and sample C: epitaxial layer with 4 × 10^14^ cm^-3^ n-type doping. The doping concentration was measured by standard mercury probe measurements. In the top row of Fig. 2, the variation of lattice spacing, Δ*d/d*, is shown for the three SiC wafers. For the SiC(0008) Bragg reflection used, the dynamical diffraction penetration depth is 5.83 µm^25,26^. The X-ray peaks that were collected are generally single peaks with very small peak widths (~ 15 µrad), and the repeatability of the goniometer is 1 µrad. Hence, for maps of sample A the diffraction peaks are from the substrate and for samples B and C the diffraction peaks are primarily from the epitaxial layers. An outline of the 150 mm SiC wafer is superimposed in order to show the regions of the wafer that were measured by the high resolution RCM measurements. From the top row of Fig. 2, it can be observed that the overall variation of lattice parameters, ∆d/d, is very small in samples A and B with a standard deviation in lattice spacing of ~ 25 µrad over the region mapped. Upon growth of a lower doped epitaxial layer, as in sample C, this variation had considerably increased with a standard deviation of 50.6 µrad, indicating the influence of doping on the lattice strain, quantitatively. We have observed that a Δd/d greater than 500 µrad can cause mechanical instability in SiC wafers that can lead to difficulties in device fabrication. The lattice strain variation can also be influenced by thermal stress present during the epitaxial layer growth. The thermal strain introduced in current state-of-art epitaxial growth systems has been vastly minimized, with thermal gradient ~ 1 °C from center of the wafer to its edge. Thus, the thermal strain introduced is minimal, and the doping variations dominate the lattice strain. Extended defects, such as dislocations, can have a small affect on lattice strain, but their overall contribution is much smaller than doping variations in current SiC wafers with dislocation densities below 10^4^ cm^-2^. The difference in doping between samples B and C was chosen to be three orders of magnitude. This was done in order to investigate the extremities of doping conditions commonly used for the drift layers of commercial SiC power devices. Typical 1.2–1.7 kV rated MOSFETs have a drift (voltage blocking) layer doping of ~ 10^16^ cm^-3^, which is closer to sample B than C. However, with the adoption of 3.3 kV MOSFETs for motor control in high speed trains^27,28^, and industrial development of 6.5 kV SiC power devices^29^ the epitaxial layer doping will have to reduce to ~ 5 × 10^14^ cm^-3^, which is closer to sample C doping. This could result in significant increase in lattice strain. The lattice strain, ∆d/d, is a compression/dilation of the crystal lattice and forms the diagonal elements of the strain tensor, whereas the LPC represents a twist/tilt in the lattice, which forms the off-diagonal elements. In SiC, which has hexagonal symmetry, the BPDs have a Burger’s vector 1/3[$11\stackrel{-}{2}0]$ or equivalent in the six fold symmetry, and they have a slip in the basal plane (0001). For the glide of such a dislocation, the diagonal elements of the strain tensor will have a higher contribution to the Peierls energy that is responsible for the BPD glide^30^. Hence, a smaller magnitude of Δd/d compared to the magnitude of LPC variation from the same region of the wafer would have higher contribution to BPD glide. The Δd/d variation, as seen in top row of Fig. 2, for samples A and B are similar and hence, BPD glide during epitaxial growth should be minimal compared to sample C, where the Δd/d variation is about 2 × higher. This is confirmed in the ultraviolet photoluminescence (UVPL) images, seen in Fig. 3, for the samples C (left) and B (right). The bright lines seen in these images are BPDs inside the epitaxial layer. It can be observed that the BPDs in the low doped sample C are more curved than in the higher doped sample B. The curvature of these lines represents BPD glide occurring during epitaxial layer growth in the basal plane. Average BPD glide in the low doped sample was calculated to be ~ 65 µm for the 10 µm thick epitaxial layer. If we assume a linear dependence of BPD glide with epitaxial layer thickness, for 30–60 µm thick epitaxial layers (for 3.3/6.5 kV devices), we could expect BPD glide occurring during epitaxial growth higher than 0.2–0.4 mm. This enhanced BPD glide could generate HLAs spanning a larger wafer region. The HLAs will generate stacking faults in this region over the entire epitaxial layer thickness, thereby degrading device reliability^31,32^. An example of BPD glide during epitaxial layer growth in a different wafer with ~ 10 µm thick epitaxial layer is shown in the UVPL image in Fig. 4. In this image, the bright lines are BPDs, and are observed to have a string of dots connected to them, which are the HLAs^33^. The HLA originates from a single BPD propagating from the substrate into the epitaxial layer, which glides during epitaxial layer growth due to Peach-Koehler forces arising from strain in the epitaxial layer^34^. The BPD glide process leaves behind short BPD segments that appear as dots leading up to the BPD line as observed. Each of these BPD segments can result in the formation of a stacking fault that expands within the device drift region. As seen in the figure, the longest BPD glided during epitaxial layer growth from below and extends over 2.5 mm in the wafer. The presence of many HLAs indicates presence of high lattice strain in this epitaxial wafer.

Hence by mapping the Δd/d component, the influence of doping can be correlated to the BPD glide during epitaxial layer growth. The lattice strain could also change its profile during device processing steps where high temperatures are involved, and the deconvolution of lattice distortions will be valuable to investigate how the epitaxial layer doping will influence BPD glide in the final devices after fabrication.

In the case of the LPC maps, shown in the bottom row of Fig. 2, the range of the color map is  ± 1,000 µrad, which is ~ 10 times larger than the Δd/d variation observed in their respective maps. The LPC maps are a gradient of the curvature in the lattice planes, where the curvature will be toroidal having components in both x and y axis. Even though the measurements were taken with the scattering plane in the [$11\stackrel{-}{2}0]$ direction, the gradient of curvature with respect to x-axis has contributions from both the x and y components. In the LPC maps, the highest variation is observed to be from the top right of the wafer to the bottom left for all the three wafers due to higher curvature in that direction. This LPC variation indicates the presence of a twist of the lattice planes along an axis that is ~ 60° from the wafer flat, which is one of the ‘*a*’ directions for SiC. The primary origin of LPC in SiC substrates is their non-equilibrium growth by physical vapor transport resulting in built-in residual strain^35–37^. The LPC maps show a small variation in sample A (substrate only) indicating a good PVT growth process. The LPC variation is observed to increase upon epitaxial layer growth for both samples B and C, similarly. The standard deviation in LPC is almost twice as large for the SiC wafers that have an epitaxial layer grown. Contrary to the local lattice strain, which is due to a local distortion inside the region, the local LPC occurs due to distortions that are forced on the region from adjacent regions. This can be seen from the schematic in Fig. 1b. The fact that LPC variation for samples B and C, in Fig. 2, are similar and higher than sample A is because the LPC in samples B and C is influenced by the interaction of the epitaxial layer with the substrate, and the LPC will likely depend on the epitaxial layer thickness, which is ~ 10 µm thick for both samples B and C. It is expected that in case of thicker epitaxial layers the LPC variation could increase. From the calculated standard deviation (σ) of the LPC variation, an average lattice bow is calculated using the relation^38^, *D* * *σ*/4, where *D* is the wafer region over which the RCM data was collected. The average lattice bow for the substrate only wafer, sample A, is calculated to be ~ 10 µm across the entire measured region. The average lattice bow calculated for both the wafers with epitaxial layers is calculated to be ~ 17 µm. We also measured the surface bow for substrate only sample A, and samples B and C after epitaxial layer growth using optical profilometry. The lattice bow increases after epitaxial layer growth, but the surface bow does not have any correlation. The surface bow across the wafer mainly depends on how the SiC substrate surface was prepared by surface polishing and does not represent the shape of the lattice planes. The LPC, which arises from the stress in the wafer, directly influences the wafer shape change. Hence, the LPC should be the critical parameter to characterize any wafer shape change issues that could cause wafer yield issues. The epitaxial layer thicknesses, doping, surface bow, and standard deviations in both Δd/d and LPC are tabulated in Table 1.

**Table 1:** Results of the measured doping, surface bow, calculated standard deviation (σ) in lattice strain and LPC, and FWHM for the three 150 mm SiC wafers.

Sample	Thickness (µm)	Doping (cm^-3^)	Surface Bow ± σ (µm)	σ (Δd/d)	σ (LPC)	FWHM Median ± σ (µrad)
A	0	3 × 10^18^	13.7 ± 5.4	25.1	269.5	14.4 ± 1.0
B	9.66	1 × 10^17^	19.5 ± 5.8	25.3	460.3	14.7 ± 2.1
C	9.71	~ 4 × 10^14^	12.5 ± 4.5	50.6	453.2	14.9 ± 2.3

The deconvolution of lattice distortions into their components of lattice strain and LPC provide quantitative information for SiC wafers and epitaxial layers that could serve as a guide for analyzing wafers that are less likely to fail during device fabrication or have reliability issues due to BPDs. These measurements would be a more accurate predictor of wafer shape changes during wafer fabrication than the wafer surface bow and warp after polishing. On other wafers that had surface bow/warp below 20 µm before device fabrication, we have observed LPC at least an order of magnitude higher than the wafers used in this work. Wafers with high LPC would present challenges in lithography during device processing.

**Figure 5:**
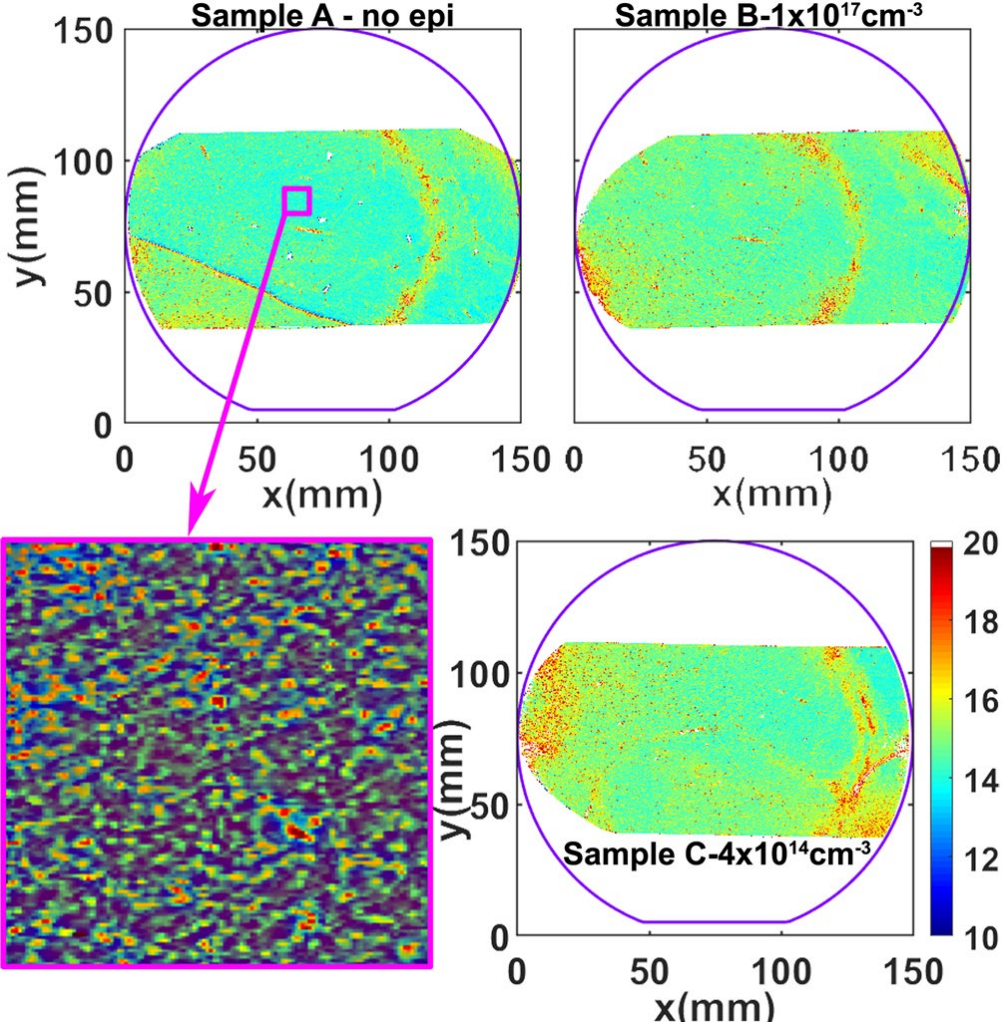
FWHM maps of the three SiC wafers with wafer outlines superimposed. The maps show dot-like contrast with higher FWHM values that are due to short range strain from threading dislocations. The sample **A** (bare substrate), shows lower density of dots compared to samples **B** and **C** (with epitaxial layer). This indicates presence of higher threading dislocations after epitaxial layer growth. The magnified view of sample A shows a 1 × 1 cm^2^ region with an average threading dislocation density of ~ 10^4^ cm^-2^. This figure was generated using 2018 Matlab software with current license purchased by the authors.

In addition to the lattice distortions, the full width at half maximum (FWHM) of the local rocking curves were mapped for all the three wafers, and are plotted in Fig. 5. The FWHM of a rocking curve is highly affected by extended defects such as dislocations. In Fig. 5 a dot like contrast is observed that is speckled across all the wafers, which arises due to the short range strain coming from threading dislocations. There are more dots in the wafers with an epitaxial layer compared to the substrate only wafer, which indicates that during epitaxial layer growth the threading dislocation density is slightly increased. At these dots the FWHM is higher, whereas the rest of the wafer has a low value between 11–15 µrad. A 1 × 1 cm region from sample A is magnified to show the dot-like contrast, which was counted to be ~ 10^4^ cm^-2^, typical of good quality SiC substrate wafer. On the right half of the wafer an arc-like region is observed with higher FWHM. This is from presence of multiple trapezoidal defects that occur in the substrate and propagate into the epitaxial layers^39,40^. These defects have a complex structure with Frank faults, BPDs and multiple double Shockley stacking faults that likely have origins from high local nitrogen doping in the substrate. The overall FWHM in sample A has a standard deviation of 1 µrad, which indicates very uniform crystalline quality in regions outside the trapezoidal defects. Upon epitaxial layer growth, the standard deviation in FWHM is doubled and also the median value is slightly increased. The median and standard deviation of FWHM are also included in Table 1.

## Conclusions

While commercial adoption of SiC based power devices is accelerating, device cost, which is directly related to yield, and device reliability that is affected by BPDs^41^, remain concerns, especially as device voltages exceed the presently available 3.3 kV devices and thicker epitaxial layers are required. High resolution X-ray rocking curve mapping was used to deconvolve the lattice distortions from Δd/d, and LPC. The Δd/d component has a higher effect on BPD glide during epitaxial layer growth. The LPC component has a stronger influence on the wafer shape change. The Δd/d variation across the wafer increases by a factor of ~ 2 for epitaxial wafer with 4 × 10^14^ cm^-3^ doping compared with the substrate or the sample with the epitaxial layer doped to 1 × 10^17^ cm^-3^. The low-doped epitaxial layer shows much more BPD glide compared with BPD glide observed in the high doped epitaxial layer. High BPD glide can lead to decreased device reliability. The LPC variation is doubled upon epitaxial layer growth irrespective of doping conditions. The LPC maps have a variation from top right to bottom left of the wafer with a twist axis that is ~ 60° from the wafer flat, indicating presence of a twist in the PVT grown wafer, which becomes higher after deposition of an epitaxial layer. From rocking curve FWHM analysis, it appears that total dislocations increase after epitaxial layer growth regardless of doping. As the demand for higher yield and reliability SiC power devices continues to grow, the high-resolution X-ray analysis presented here provides a superior method for assuring that the quality of SiC wafers does not hinder that growth.

## Methods

**Figure 6:**
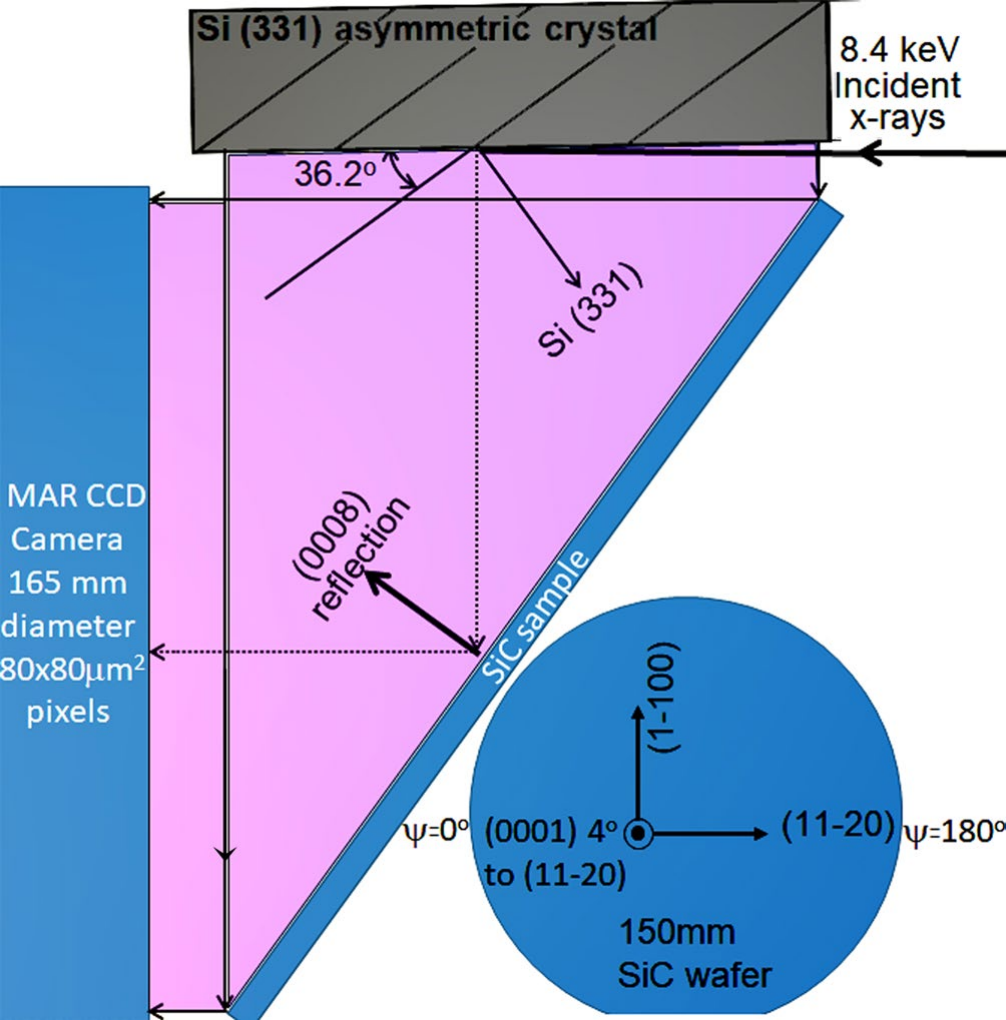
Schematic of the experimental setup at 1BM beamline at the ANL synchrotron. This figure was generated in 2016 Microsoft Powerpoint by the authors. The authors own a license.

For this study, we obtained 3 adjacent 150 mm substrates from the same boule. This provides substrates with similar starting strain conditions and extended defect densities for comparison. Epitaxial layer growth was performed using chemical vapor deposition on 2 wafers for an epitaxial layer thickness of ~ 10 µm, and nitrogen doped to ~ 4 × 10^14^ cm^-3^ (sample C), and 1 × 10^17^ cm^-3^ (sample B). The third substrate (sample A) was kept as a reference. The epitaxial layer was grown on samples B and C without any buffer layers in order to observe effects of abrupt change in doping from substrate to the epitaxial layer. The strain contributions on these substrates with and without 10 µm thick epitaxial layers were measured using ultra-high resolution rocking curve mapping (RCM) technique at the 1BM beamline of the Argonne National Laboratory (ANL). Figure 6 shows the schematic of the setup, where the incident X-ray beam was set to have energy of 8.4  ± 0.0005 keV using water cooled Si (111) double crystal monochromators. A 100 mm diameter nearly perfect Si crystal was used as a beam conditioner. This crystal was asymmetrically cut 36.2  ± 0.1° from Si (331) plane, which provides horizontal magnification of the incident beam in order to illuminate a large SiC wafer area. The Si (331) Bragg reflection is also ~ 0.5° difference from the SiC (0,008) Bragg reflection, so the dispersion is greatly minimized in order to measure small strains on the order of 10^–6^ radians. The 1/e depth from dynamical diffraction theory for the SiC (0,008) reflection is calculated to be 5.83 µm. The SiC wafers were mounted and aligned on a highly precise Kohzu goniometer with angular repeatability of 1 µrad. In the arrangement, we obtained a large incident X-ray beam footprint that covers ~ 150 mm × 90 mm, which enabled the measurement from a large area of the 150 mm SiC wafer. A 165 mm diameter CCD detector having 80 × 80 µm^2^ pixels was used to collect the Bragg reflections. The SiC wafers were aligned in the (0008) reflection and RCM with a step of 4 µrad were recorded over the range of ~   ± 1,000 µrad. The images were taken at two azimuthal conditions rotated by 180°. This was performed in order to deconvolve the strain in the wafers into the contributions from lattice spacing variations (Δd/d), and lattice plane curvature (LPC). Fiducial marks were applied on the wafers so that the same regions of the wafer could be paired with each other after 180° rotation. The strain deconvolution was performed by first fitting the local rocking curve in each camera pixel to extract the peak position and width at that pixel. The peak shifts from the two azimuthal conditions, which are mutually 180° apart, are added, and subtracted using Eqs. (2) and (3) to obtain the Δd/d and LPC strain contributions.
